# Ethylmalonic Encephalopathy ETHE1 R163W/R163Q Mutations Alter Protein Stability and Redox Properties of the Iron Centre

**DOI:** 10.1371/journal.pone.0107157

**Published:** 2014-09-08

**Authors:** Bárbara J. Henriques, Tânia G. Lucas, João V. Rodrigues, Jane H. Frederiksen, Miguel S. Teixeira, Valeria Tiranti, Peter Bross, Cláudio M. Gomes

**Affiliations:** 1 Instituto de Tecnologia Química e Biológica António Xavier, Universidade Nova de Lisboa, Oeiras, Portugal; 2 Research Unit for Molecular Medicine, Aarhus University Hospital, Aarhus, Denmark; 3 Unit of Molecular Neurogenetics, Foundation IRCCS Neurological Institute Carlo Besta, Milan, Italy; University of South Florida College of Medicine, United States of America

## Abstract

ETHE1 is an iron-containing protein from the metallo β-lactamase family involved in the mitochondrial sulfide oxidation pathway. Mutations in ETHE1 causing loss of function result in sulfide toxicity and in the rare fatal disease Ethylmalonic Encephalopathy (EE). Frequently mutations resulting in depletion of ETHE1 in patient cells are due to severe structural and folding defects. However, some ETHE1 mutations yield nearly normal protein levels and in these cases disease mechanism was suspected to lie in compromised catalytic activity. To address this issue and to elicit how ETHE1 dysfunction results in EE, we have investigated two such pathological mutations, ETHE1-p.Arg163Gln and p.Arg163Trp. In addition, we report a number of benchmark properties of wild type human ETHE1, including for the first time the redox properties of the mononuclear iron centre. We show that loss of function in these variants results from a combination of decreased protein stability and activity. Although structural assessment revealed that the protein fold is not perturbed by mutations, both variants have decreased thermal stabilities and higher proteolytic susceptibilities. ETHE1 wild type and variants bind 1±0.2 mol iron/protein and no zinc; however, the variants exhibited only ≈10% of wild-type catalytically activity. Analysis of the redox properties of ETHE1 mononuclear iron centre revealed that the variants have lowered reduction potentials with respect to that of the wild type. This illustrates how point mutation-induced loss of function may arise via very discrete subtle conformational effects on the protein fold and active site chemistry, without extensive disruption of the protein structure or protein-cofactor association.

## Introduction

In many inborn errors of metabolism pathological genetic variations resulting in loss of protein function are frequently categorized as catalytic versus structural mutations, as they relate respectively to effects on biological function or on protein folding and stability. Whereas the latter can be more or less accurately assigned from absence of protein in patient-derived tissues, the so called catalytic mutations frequently include very significant conformational flaws which actually end up driving to loss of function by diverse molecular mechanisms. There are numerous mutations affecting diverse metabolic pathways that fall in a grey area in which both catalysis and structure are affected to a moderate, but yet deleterious, extent. It is therefore important to collect comprehensive data on such disease-related variants to gather a deeper understanding into the molecular mechanisms that underlie loss of function in inborn errors of metabolism as well as to broaden the scope of classifications of different types of mutations.

Genetic lesions in the *ETHE1* gene are associated to ethylmalonic encephalopathy (EE), a devastating infantile metabolic disorder affecting the brain, gastrointestinal tract, and peripheral vessels [1]. EE is recessively inherited and mutations are usually causing loss of function in ETHE1, an important enzyme in the mitochondrial sulfide catabolic pathway. Hydrogen sulfide (H_2_S) is a water-soluble molecule, gaseous at ambient pressure and temperature, which functions as a signaling molecule, but is toxic at high concentrations [2], [3], similarly to several gasotransmitters [4]. In humans, sulfide is synthesized from L-cysteine that is taken up in the diet or synthesized by catabolic and anabolic processes. A catabolic pathway oxidizing sulfide to the major end-product sulfate or sulfate esters and taurine has recently been elucidated [5]. Moreover, ETHE1 was identified as a sulfide dioxygenase that catalyzes one reaction in this catabolic pathway. ETHE1 is a member of the metallo β-lactamase enzyme family, which harbor mononuclear or binuclear metal centres containing one or two iron and/or zinc atoms [6], [7]. The structure of human ETHE1 has not been determined experimentally, and modeling as well as experimental investigations have led to conflicting suggestions in what concerns the stoichiometry and type of redox centre present in ETHE1 [8], [9].

In the majority of cases, mutations lead to absence of ETHE1 protein due to partial gene deletion, degradation of premature-termination codon containing ETHE1 transcripts by nonsense mediated decay, or by degradation of misfolded ETHE1 proteins by quality control proteases [10]. However, in a few cases, ETHE1 has been detected in patient cells which display a clinical phenotype indistinguishable from that of ETHE1 protein-negative cases, suggesting that in such circumstances the mutant protein lacks activity. Two such cases are those of the disease-associated ETHE1 mutations, found in homozygous EE patients, that both affect arginine-163, replacing it by tryptophan or glutamine [1], [9], [11]. Patient fibroblasts were protein positive but since these individuals were affected by EE, arginine 163 has been suggested to be critical for ETHE1 catalytic activity. In this work we address the molecular mechanisms through which these mutations give rise to disease. Using a combination of biochemical and biophysical methods we establish a set of basic features of the wild type protein and analyze the extent to which disease-associated variations of arginine 163 affect ETHE1 structure and activity.

## Materials and Methods

### ETHE1 expression and purification

*E. coli competent cells* Bl21 DE3, from Novagen, transformed with the respective plasmid, pMW172ETHE1 wild type, pMW172ETHE1-p.Arg163Trp, or pMW172ETHE1-p.Arg163Gln, all with a 3′ sequence coding region for six histidines [9] were grown in dYT medium (16 g Bacto Tryptone, 10 g Bacto Yeast extract and 5 g NaCl) supplemented with 100 µg.ml^−1^ ampicillin and 10 µM FeSO_4_ at 37°C in a shaking incubator until OD_532_ of 0.5–0.8 was reached. The cells were then induced overnight with 0.1 mM isopropyl-1-thio-β-D-galactopyranoside (IPTG) at 25°C. Cells were harvested by centrifugation, re-suspended in 50 mM Tris-HCl pH 7.5, 300 mM NaCl, 10 mM imidazole and 0.5 mM phenylmethylsulphonylfluoride (Roth) (buffer A) in presence of DNase (Applichem) and disrupted in a French press. The soluble extracts were applied on His-trap HP columns (GE Healthcare, 5 ml) previously equilibrated in buffer A. The columns were washed with 5 volumes of buffer A, and bound proteins were eluted by a linear gradient of 10–500 mM imidazole in buffer A. ETHE1 eluted at an imidazole concentration around 180 mM. Further purification was done by size exclusion chromatography (Superdex 75, GE Healthcare) with 50 mM Tris-HCl pH 7.5, 150 mM NaCl. The column was previously calibrated using standard proteins, and the native molecular mass of ETHE1 was determined. The purity of preparations was confirmed by SDS/PAGE and protein concentration was determined using the Bradford assay.

### Determination of metal content

Iron content of the ETHE1 wild-type and variant proteins was determined using the TPTZ (2,4,6-tris (2-pyridyl)-S-triazine) method [12]. A calibration curve was done using accurately titrated Fe(NO_3_)_3_ solutions (a kind gift from Rita Delgado, ITQB/UNL). Positive controls of 1 and 2 nmoles of the *A. ambivalens* [3Fe-4S][4FE-4S] Ferredoxin [13], [14], [15] were analyzed together with 5 nmoles of ETHE1 proteins. The proteins were diluted in milliQ water before denaturation with hydrogen chloride, and left on ice for 10 minutes. Trichloroacetic acid was used to precipitate the denatured proteins and the solutions were centrifuged for 15 minutes at 13000 rpm. Ammonium acetate and hydroxylammonium chloride were added to 800 µl of the supernatants. TPTZ was added immediately before recording the absorbance at 593 nm. The amounts of zinc and copper were determined using a modified Zincon-based protocol described in the literature [16]. Calibration curves were done using known concentrations of ZnSO_4_ and CuSO_4_. Boric acid buffer with a final concentration of 8 M urea and 50 mM borate at pH 9.0 was used, as well as 1 mM Zincon solution. The spectrum from 700 nm to 450 nm was recorded and the absorbance at 620 nm used to quantify the amount of total metal in the sample. In parallel EDTA was added to the samples to remove zinc that would bind to Zincon. Analysis of the absorbance at 620 nm, after EDTA was added, was used to determine the amount of copper in the sample. Subtracting the amount of copper from the total amount of metal in the solution, one determined the amount of zinc in solution. ETHE1 protein (1.3 nmol) was used in the analysis and positive controls of 2.5 nmol hHolo SOD1 (1Cu:1Zn per mol) and Ferredoxin (7Fe:1Zn per mol) were used, as well as a negative control of 2.5 nmol hApo SOD1 (fully demetallated). To release any protein-bound metals, the samples were incubated with 0.2 M HCl and subsequently neutralized using 0.2 M NaOH and the pH adjusted to 9. For all determinations at least three measurements were made.

### Enzymatic assays

ETHE1 activity was determined according to its capacity to oxidize glutathione persulfide [5], [17], in an assay adapted from [5]. Briefly, enzyme activity was measured using a Hansatech DW1 oxygen electrode with a magnetic stirrer at room temperature and following O_2_ consumption in an assay where buffer 0.1 M KPi pH 7.4, saturated acetonic sulfur solution, 1 mM reduced glutathione and ETHE1 were employed. Saturated acetonic sulfur solution and reduced glutathione were used to form the glutathione persulfide. One unit of catalytic activity is defined as µmol of O_2_ consumption per minute, at room temperature.

### Circular Dichroism

Far-UV CD spectra were recorded on a Jasco J-815 spectropolarimeter with a cell holder thermostatically controlled with a Peltier element. A quartz polarized 1 mm path length quartz cuvette (Hellma) was used, and typical protein concentrations were 0.1 mg.ml^−1^ in 50 mM Tris-HCl pH 7.5, 150 mM NaCl.

### Protein thermal stability

Thermal unfolding with a linear temperature increase at 1°C.min^−1^, from 25 to 90°C, was followed using circular dichroism (ellipticity variation at 222 nm). The protein concentration was 0.1 mg.ml^−1^ in 50 mM Tris-HCl pH 7.5, 150 mM NaCl. Data were analyzed according to a two-state model, and fits to the transition curves were made with OriginPro8. The temperatures at the midpoint transition are an average of three independent determinations.

### Trypsin limited proteolysis

Digestion of ETHE1 recombinant proteins, wild type and p.Arg163Gln and p,Arg163Trp variants, was performed by incubating the proteins with trypsin (bovine pancreas trypsin, Applichem), at 37°C in 0.1 M Tris-HCl pH 8.5, in a 10-fold excess over the protease. As a control, identical samples without addition of trypsin were also subjected to the same procedure. Aliquots with 0.05 nmol of protein were sampled at different time points up to 1 h, and the reaction was stopped by the addition of SDS-PAGE loading buffer (2% SDS and 5% β-mercaptoethanol). The products of the proteolysis reaction were analyzed by 12% SDS/PAGE, stained with Coomassie Brilliant Blue R-250. As an internal standard for the quantity of loaded protein in the gel slots, bovine serum albumin (BSA, Sigma), 2.5 µM final concentration, was also added to loading buffer solution.

### Electron Paramagnetic Resonance

EPR spectra were collected on a Bruker EMX spectrometer equipped with an ESR 900 continuous-flow helium cryostat from Oxford Instruments and were recorded at 9.39 GHz microwave frequency, 2.0 mW microwave power, 1 mT modulation amplitude, at 8 K.

### Redox titration

Redox titrations followed by EPR spectroscopy were performed anaerobically at 25°C under an argon atmosphere. The reaction mixture contained ETHE1 at 1 mg.mL^−1^, 150 mM Tris-HCl pH 7.5, 300 mM NaCl, and a mixture of the following redox mediators, each at 80 µM concentration, indigo tetrasulfonate (−130 mV), 2-hydroxy-1,4-naphtoquinone (−152 mV), anthraquinone-2-sulfonate (−225 mV), phenosafranine (−255 mV), safranine (−280 mV), neutral red (−325 mV), benzyl viologen (−345 mV), methyl viologen (−440 mV). Glucose oxidase (7.5 U.mg^−1^), glucose (6 mM) and catalase (15 U.mg^−1^) were also included in the solution to consume traces of oxygen. Buffered sodium dithionite (250 mM Tris-HCl, pH 9.0) was used as reductant. The reduction potential measurements were performed using platinum and Ag/AgCl chloride electrode, calibrated with a quinhydrone saturated solution at pH 7.0. The reduction potentials were determined by fitting a Nernst equation for a single one-electron transition, and are quoted against the standard hydrogen electrode.

## Results

### ETHE1-p.Arg163Gln and p.Arg163Trp are folded but have decreased stability

**Figure 1:**
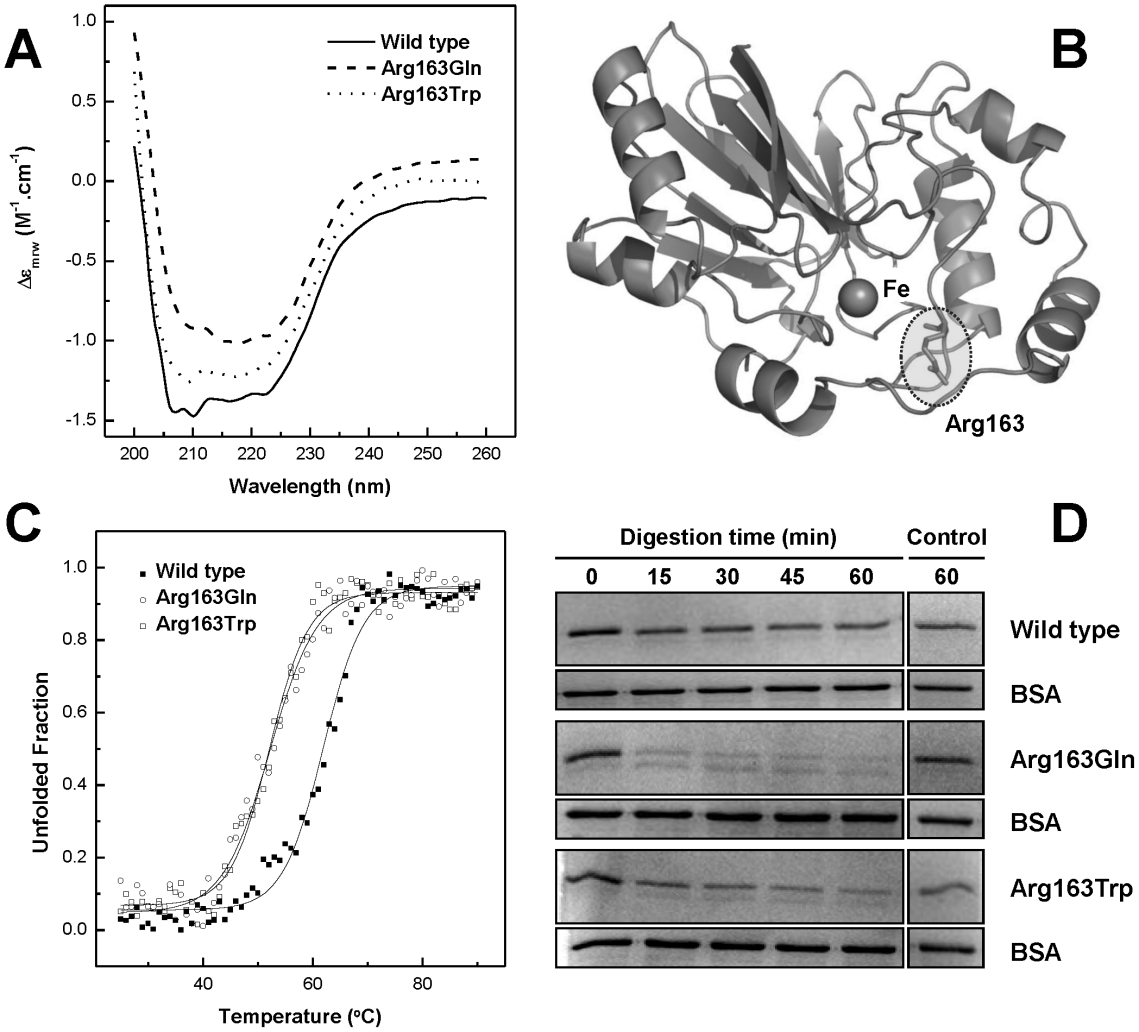
ETHE1-p.Arg163Gln and p.Arg163Trp are folded but have decreased stability. **A**. Far-UV CD spectra of purified ETHE1 proteins at 0.1 mg.ml^−1^ in 50 mM Tris-HCl pH 7.5, 150 mM NaCl. Spectra are offset for clarity. **B**. Molecular model of human ETHE1 highlighting the position of Arg163 in respect to the Fe centre, molecular modeling was made based in the *Arabidopsis thaliana* ETHE1-like protein structure [18]. **C**. Thermal denaturation curves of ETHE1 proteins, followed by ellipticity variation at 222 nm from which the fraction of unfolded protein is determined. **D**. Time course of limited proteolysis experiment at 37°C in 0.1 M Tris-HCl, pH 8.5 monitored by SDS-PAGE. See Materials and Methods for details.

Human ETHE1 proteins, wild type and disease-associated variants, were expressed in *E. coli* and purified to homogeneity (>95%). Analysis of the quaternary structure of the purified proteins by size exclusion chromatography showed that they exist in solution as dimers (data not shown), as previously reported [17], [18]. In order to define the structural characteristics of ETHE1 wild-type and variants, we have investigated the overall ETHE1 fold and conformational properties using circular dichroism (CD) spectroscopy (Fig. 1A). The far-UV CD spectra showed a clear α-helix fingerprint with minima at 208 and 222 nm, which are typical of well folded α/β proteins. A comparison of the CD spectra of the ETHE1 wild type protein and the mutant proteins, p.Arg163Gln and p.Arg163Trp, shows superimposable features, indicating that these mutations do not disrupt the protein fold or its secondary structure (Fig. 1A). Indeed, molecular modelling on the basis of the *Arabidopsis thaliana* ETHE1-like structure (54% amino acid identity, [18]) is consistent with human ETHE1 adopting a β-lactamase-like fold (Fig. 1B), characterized by a core unit of two β-sheets sandwich flanked by two sets of solvent exposed α-helices. Replacement of Arg163 localized in a peripheral loop connecting two beta turns is not expected to largely perturb the protein structure, in agreement with our CD measurements. However, inspection of the *A. thaliana* ETHE1 structure [18] shows that Arg163 side chain is relatively proximal to Fe (≈10 Å) and that H-bonding with a contiguous conserved residue (Asp165) may indicate a role in the organization of the loop adjacent to the metal binding site.

We then investigated if any of the mutations would affect protein stability. For the purpose we monitored the enzyme secondary structure using CD spectroscopy as a function of temperature. We have found that all proteins undergo temperature-induced aggregation, that wild-type ETHE1 unfolds at an apparent midpoint unfolding temperature (T_m_) of 62°C, and that ETHE1-p.Arg163Trp and ETHE1-p.Arg163Gln exhibit a significant destabilization with lower T_m_ values of 53 and 55°C, respectively (Fig. 1C, Table 1). These differences were further corroborated by the results of the proteolytic susceptibility of the wild type ETHE1 protein in comparison to those of the disease-associated variants. The rationale for this approach is that a destabilized conformation will have a higher number of cleavable sites accessible to digestion, as a result of a higher flexibility of the polypeptide chain [19]. Indeed, a time course analysis evidenced a decreased stability of the mutant variants towards proteolysis: under the tested conditions wild type ETHE1 is mildly digested, while the pathological variants are degraded to significantly higher degree (Fig. 1D).

**Table 1:** Biochemical and conformational properties of ETHE1.

	ETHE1-WT	ETHE1-p.Arg163Trp	ETHE1-p.Arg163Gln
**Specific Activity** (U.mg^−1^)	21.0±3.0	1.8±0.9	2.4±0.9
**Iron content** (mol Fe/mol protein)	1.1±0.1	1.2±0.1	1.2±0.3
**Thermal stability** T_m_ (°C)	62±2	52±2	55±2
**Reduction Potential** E^0^ (mV)	−272±15	−370±15	−310±15

The values presented for the different properties correspond to an average of at least three independent determinations (n = 3).

### ETHE1-p.Arg163Gln and p.Arg163Trp affect the mononuclear iron site reduction potential

Previous reports of loss of function by the ETHE1-p.Arg163Gln and p.Arg163Trp disease-associated variants have not addressed the molecular mechanisms that underlie the disease-causing nature of these variants [9]. Having established that the mutant proteins have a native-like structure, which rules out protein misfolding as the origin of functional deficiency, we then investigated if any of these mutations affect metal binding. A systematic analysis of the metal content in the purified wild type and mutant ETHE1 variants showed the absence of zinc and the presence of iron at a molar ratio close to 1 (Table 1). These results show that our strategy encompassing expression of ETHE1 proteins with Fe-supplementation resulted in a fully loaded mononuclear Fe centre in the protein. Nevertheless, in spite of a fully loaded Fe site and properly folded structure, enzymatic activity measurements revealed that ETHE1-p.Arg163Gln and p.Arg163Trp display compromised catalytic rates, with activities around 10% of the wild type (Table 1).

**Figure 2:**
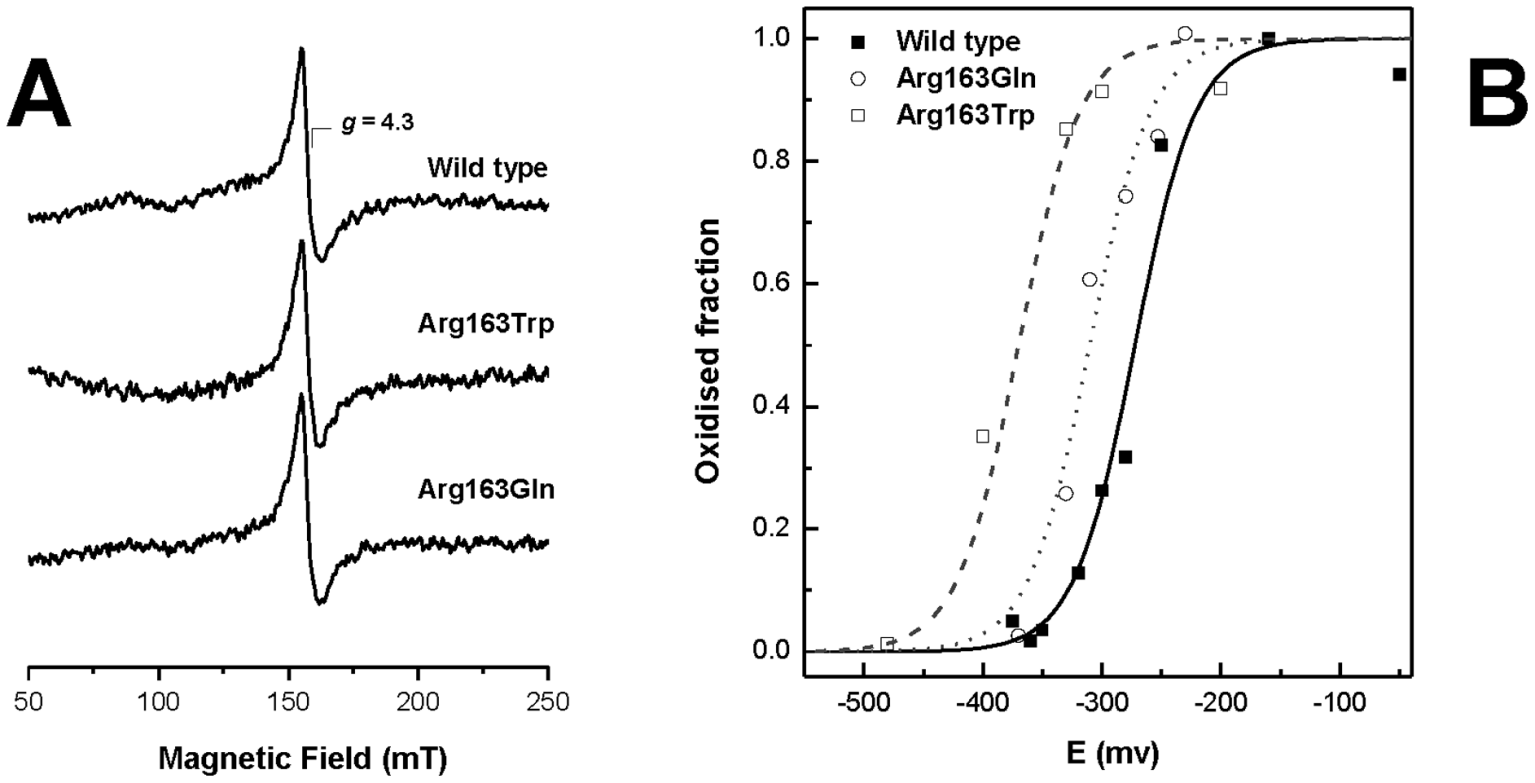
ETHE1-p.Arg163Gln and p.Arg163Trp mutations affect the mononuclear iron site reduction potential. **A**. EPR spectra of ETHE1 proteins at 1 mg.ml^−1^ in 150 mM Tris-HCl pH 7.5 and 300 mM NaCl. **B**. Redox titrations followed by EPR spectroscopy. Solid and dashed lines are Nernst Curves obtained for the different ETHE1 proteins one-electron reduction processes (n = 1) and the oxidation-reduction potentials are listed in Table 1. EPR spectra were recorded at 9.39 GHz microwave frequency, 2 mW microwave power, 1 mT modulation amplitude, 8 K temperature.

We then investigated if any of these mutations in ETHE1 could compromise the properties of the mononuclear Fe centre, especially in what concerns its oxidation-reduction properties which naturally determine the reactivity of ETHE1 as a sulfur dioxygenase. For this purpose we used Electron Paramagnetic Resonance (EPR) spectroscopy to analyze the properties of ETHE1 iron centre. The EPR spectrum of the wild type ETHE1 and variants, ETHE1-p.Arg163Gln and p.Arg163Trp, shows a signal at *g* = 4.3 arising from the middle (|Ms = ±3/2>) Kramers doublet of a high-spin (S = 5/2) ferric species with rhombic distortion (maximum rhombicity, E/D, of ∼0.33), which is compatible with a mononuclear iron site in a weak ligand field, and agrees with our metal analysis data (Fig. 2A). We then used this spectroscopic fingerprint to determine the reduction potential of the ETHE1 iron centre, as this signal disappears upon reduction of iron from the ferric to the ferrous state. An EPR-monitored redox titration was then performed by poising ETHE1 proteins at different redox potentials from which the intensity of the *g* = 4.3 signal was determined (Fig. 2B, Table 1). From this analysis, the reduction potential (*E*°) of wild type ETHE1 was found to be −272 mV (±15 mV), whereas a significant lowering of the reduction potentials was measured for ETHE1-p.Arg163Gln and p.Arg163Trp (*E*° = −310 mV and −370 mV, (±15 mV) respectively). This change in the reduction potential, along with subtle structural reorganization of the active site electrostatics that most likely affects substrate binding, results in decreased activity.

## Discussion

Missense mutations resulting in protein loss of function in metabolic disease are frequently labeled as catalytic or structural mutations, depending whether they affect protein function or folding. However, this simplistic dichotomy can be misleading as protein structure and function are interrelated and the change of a single amino acid harnesses the potential to affect both. Here we report an investigation on the effects of the disease-associated ETHE1 mutations p.Arg163Trp and p.Arg163Gln. These pathological variants are found in EE patients and we used recombinant human ETHE1 proteins to address how these changes influence the catalytic properties and conformational stability of the protein, using a series of biophysical and biochemical assays.

Our study confirms that ETHE1 is a dimeric protein [17], [18] and establishes that it contains one iron per subunit, in contrast to previous reports that indicated a lower stoichiometry [17]. Also, we show that ETHE1 does not bind zinc, suggesting the presence of a mononuclear iron site in ETHE1, a feature which has so far remained relatively unclear in the previous literature. It is well known that recombinant expression of metalloproteins may result in conformers with poorly assembled metal centres unless adequate metallochaperones are simultaneously co-expressed, or the growth medium is supplemented with the adequate metal in a bioavailable form. In this study, and to minimize substoichiometric metal binding to ETHE1, protein expression was always carried out in Fe-supplemented medium which resulted systematically in protein preparations which contain ≈1 mol Fe/mol protein, indicating the presence of a mononuclear Fe site in ETHE1. The simultaneous analysis of iron and zinc was a very important control, as proteins with the metallo-β lactamase fold are known to be able to harbor binuclear metal sites (Fe-Fe or Fe-Zn) [7], [20]. Our results thus clarify this aspect of ETHE1 biochemistry and the determined specific activity of 21±3 U.mg^−1^ thus constitutes the updated reference value for ETHE1 sulfur dioxygenase activity.

With respect to structural and stability effects, both mutant variants are compromised in their conformational stabilities. ETHE1-p.Arg163Trp and ETHE1-p.Arg163Gln present T_m_ values lower than those of the wild type form (T_m_≈52°C versus T_m_≈62°C) but yet retain the same fold and relative secondary structure composition at 37°C. However, mutations do impact on ETHE1 breathing dynamics as the pathological variants have an increased sensitivity to proteolytic degradation; nevertheless this susceptibility is insufficient to result in decreased steady state levels in patient fibroblasts that could explain the clinical phenotype [9]. In agreement, these perturbations do not affect the formation of the mononuclear iron site, as both purified variants also contain ≈1 mol Fe/mol protein (Table 1). However, the catalytical properties of these two disease-associated variants are severely impaired. Our investigation of the reduction potential of the iron centre using EPR spectroscopy showed that the mononuclear iron sites in the mutant enzymes have lowered reduction potential values, which makes oxidative reactions less favourable. Indeed, analysis of a molecular model of human ETHE1 [9] suggests that removal of the positively charged side chain from Arg163, which is in the proximity of the active site, makes acceptance of one electron by the iron atom less favorable, resulting in a decrease in the reduction potential, which is precisely the outcome we observed experimentally. Moreover, although the ETHE1-p.Arg163Trp mutant present Δ*E*° of −100 mV in respect to the wild type, and the ETHE1-p.Arg163Gln mutant has only a variation of −40 mV, both mutants present the same degree of impaired activity. This could be an indication that a small variation of the reduction potential could be sufficient to abolish enzymatic activity, albeit other yet unknown factors may also play a role.

In conclusion, the ethylmalonic encephalopathy ETHE1-p.Arg163Gln and p.Arg163Trp mutations influence protein stability and the redox properties of the iron centre in this mitochondrial matrix metalloprotein. The paradox in this case is that these ETHE1 catalytic mutations which lead to adequately folded protein, are found in patient fibroblasts and have a properly inserted iron cofactor. Yet, their slight destabilization and increased dynamics, along with a chemically different side chain in a position relatively close to the active site, alters the bonding network and electrostatic environment around the mononuclear Fe centre, affecting its reduction potential. These structural rearrangements reshape the electrostatic environment at the ETH1 reactive site and this is also likely to affect protein-substrate interactions, eventually mediated by Arg165, thus also contributing to decreased activity. Herein these subtle conformational changes lay a possible mechanistic explanation for ETHE1 loss of activity and function. In comparison to ‘catalytic’ mutations (which disrupt protein-cofactor association, the active site geometry or substrate binding pocket) and to ‘structural’ mutations (which result in misfolding and cellular protein degradation), this is an example of a mixed effect with a strong active site jeopardizing element and eventually these types of clinical variations could be more accurately referred to as ‘mixed catalytic-structural’ mutations. It is noteworthy that such mixed effects have also been identified in other inborn errors of metabolism, that for instance include the ETF variants p. βAsp128Asn and p. βArg191Cys associated with mild cases of multiple acyl-CoA dehydrogenation deficiency (MADD) [21], [22]; the MCAD p.Thr193Ala variant with altered catalytic activity, folding and stability associated with MCAD deficiency [23], [24]; and two polymorphic SCAD variants, p. Gly185Ser and p. Arg147Trp, which are susceptible to present a decreased activity and thermal stability [25]. Therefore, we emphasize the importance to consider combined effects when analyzing disease causing mutations in inborn errors of metabolism, and we put forward the suggestion to use the designation ‘mixed catalytic-structural variants’ to classify these cases.
